# Comparative Approach to Non-Traumatic Acute Abdominal Pain Between Elderly and Non-Elderly in the Emergency Department: A Study in Rural Greece

**DOI:** 10.4021/jocmr1424w

**Published:** 2013-06-21

**Authors:** Apostolos Pappas, Hariklia Toutouni, Stavros Gourgiotis, Charalampos Seretis, Ilias Koukoutsis, Ioannis Chrysikos, George Gemenetzis, Ioannis Matzoukas, George Karavitis, Emmanouil Lagoudianakis

**Affiliations:** a Second Surgical Department, NIMTS Hospital, Athens, Greece; b Department of Nursing, A’ Technological Educational Institute of Athens, Greece; c Second Surgical Department, 401 General Army Hospital of Athens, Greece

**Keywords:** Approach, Abdominal pain, Elderly, Non-elderly, Emergency department

## Abstract

**Background:**

Acute abdominal pain is one of the most common symptoms that emergency department physicians encounter during their practice. The difficult task of early diagnosis and management of abdominal pain becomes more complicated when it involves elderly patients. The aim of this study was to evaluate the presence of age based differences regarding the management of acute non-traumatic abdominal pain in the Emergency Department.

**Methods:**

We retrospectively analyzed the medical records of 933 patients with acute non-traumatic abdominal pain in the emergency department of a regional hospital during one year period.

**Results:**

There were no differences between native and foreign elder patients regarding the use of imaging studies and discharge status. Although no differences were detected regarding the clinical presentation and management within the Emergency Department, elder patients with abdominal pain had a higher likelihood of being admitted for further hospitalization and were more often submitted to diagnostic examinations. The elder group had a trend towards lower number of cases of non-specific abdominal pain in comparison with the non-elders. Between male and female elders no statistically significant differences were detected.

**Conclusions:**

A thorough work-up is essential for all patients. The clinician should always be alerted, since elderly patients may require more tests and they should have a low threshold for hospital admission.

## Introduction

Acute abdominal pain is a non-specific symptom of many diseases. It remains one of the most common reasons that patients complain in the Emergency Department (ED). It accounts for 5-10% of all ED patients [1-3]. It can be one of the symptoms, associated with transient disorders or an abdominal disease that may require surgical intervention [4, 5]. Furthermore, emergency physicians need to be well-versed in its management, particularly in dealing with acute pain.

The elderly (aged 65 years and older) represent the fastest growing subset of population. It is estimated that one fourth of the patients, complaining for acute abdominal pain in the ED, are older than 50 years [6, 7]. These patients represent a significant and challenging diagnostic problem due to the atypical presentation of their symptoms, the presence of coexisting diseases, the atypical physical examination findings and laboratory values, and the higher morbidity and mortality they have [7-9]. Early diagnosis and proper treatment are essential to prevent further deterioration of their health status. The latter is likely to be caused even in more benign conditions due to their weakened immune system [10].

A limited number of studies have demonstrated differences in the diagnostic evaluation of abdominal pain between elderly and non-elderly patients [11]. The aim of this study was to assess the presence of differences in clinical presentation and management among non-elderly and elderly patients presenting with acute non-traumatic abdominal pain. Our study is the first to attempt to investigate the extent of the differentiations in terms of identifying potential differences regarding admission characteristics, diagnostic work-up and referral rates for hospitalization of non-traumatic abdominal pain in the elderly, compared to non-elderly patients, within the framework of an Emergency Department in a rural hospital of a National Health System, in which the specialty of “geriatrics” is not formally recognized and the management of elderly patients is performed by doctors with various levels of experience and familiarization with elderly medicine.

## Materials and Methods

This retrospective study was conducted between January 2010 and January 2011 in the ED of a regional hospital of Greece. This province is close to the capital and its current population coverage is approximately 106,000 people, which increases very rapidly during weekends and summer months. The study protocol was approved by the Hospital’s Ethical Committee.

The medical records of 933 patients, who presented to the ED with a current chief complaint or an ED diagnosis of acute non-traumatic abdominal pain, were reviewed. We arbitrarily chose 65 years as our age cutoff between elder and non-elder in order to be consistent with other published studies. Patients younger than 16 years, with abdominal trauma, as well as patients with incomplete charts were not included in this study.

Data regarding the patients’ age, gender, presence of health insurance, and nationality was noted and analyzed. We also recorded findings of physical examination, such as the presence of fever, tachycardia, signs of peritonitis, and symptoms duration prior to patients’ presentation at the ED. Data analysis also included results from laboratory investigations, the necessity to perform plain abdominal x-ray or abdominal ultrasound (U/S) or computerized axial tomography (CAT) scan, and the treatment modalities used in the ED. Patients were either discharged with medical prescriptions or admitted to the Internal Medicine or Surgical Departments for further management.

### Statistical analysis

Data were entered into a database and analyzed using the Statistical Package for the Social Sciences version 16.0 (SPSS, Chicago, Ill, USA). Descriptive statistics were calculated for all variables. Categorical variables were analyzed with the chi-square test or Fisher’s exact test as appropriate. The one-sample Kolmogorov-Smirnov test was used to test if a variable was normally distributed. All data were normally distributed and they are presented as mean ± SD. The independent sample t-test was used to detect differences in the mean hematocrit (Ht) and the length of stay between elders and non-elders. Probability (P) values less than 0.05 were considered statistically significant.

## Results

The current study sample consisted of 413 male and 520 female patients with a mean age of 42.3 ± 21.6 years. Twenty four percent of our patients aged more than 65 years. In the elderly group, 99% were native and also had health insurance. The patients located their pain mainly in the epigastrium (25%) and hypogastrium (35%). Eighty three percent of the patients presented within 24 hours after the onset of their symptoms, 11% were febrile, 25% tachycardic, 43% had signs of peritonitis, and 33% presented with leukocytosis. All patients received abdominal x-ray, 35% abdominal U/S, and 5% abdominal CAT scan. Concerning the medications administered at the Emergency Department, 63% of the patients received proton pump inhibitors (PPIs) or H_2_ receptor antagonists, 23% received analgesics and 13% a combination of both. Moreover, 43% of patients were admitted for further evaluation and 8% were in need of surgical intervention.

The elder group included a higher percentage of native patients (99% vs. 79%) with health insurance (99% vs. 86%) compared to non-elders (P < 0.05). Statistical analysis revealed no differences between native and foreigner elder patients regarding the use of imaging studies and discharge status. Between elder and non-elder patients no differences were detected with respect to site and duration of abdominal pain (Table 1).

**Table 1:** Demographic Data and Clinical Findings Stratified by Age

	< 65	≥ 65	P-value
Nationality			0.00
Native	79%	99%	
Foreigner	21%	1%	
Health insurance			0.00
No	14%	1%	
Yes	86%	99%	
Gender			0.07
Male	42%	49%	
Female	58%	51%	
Pain location			0.08
Located	94%	97%	
Diffuse	6%	3%	
Symptoms’ duration (hours)	25.8 ± 27.6	21.1 ± 21.4	
< 24	74%	83%	0.41
≥ 24	26%	17%	0.11
Fever			0.07
No	95%	89%	
Yes	5%	11%	
Tachycardia			0.46
No	81%	75%	
Yes	19%	25%	
Signs of peritonitis			0.96
No	57%	57%	
Yes	43%	43%	

Laboratory values did not significantly differ between the two groups and the patients received similar treatment modalities (Table 2).

**Table 2:** Blood Tests, Imaging Studies and Treatment Modalities Stratified by Age

	< 65	≥ 65	P-value
Leukocytosis			0.46
No	71%	67%	
Yes	29%	33%	
Hematocrit (%)	42 ± 5	42 ± 4	0.91
Ultrasound			0.01
No	78%	65%	
Yes	22%	35%	
CT scan			0.00
No	99%	95%	
Yes	1%	5%	
PPI/H2 antagonists	63%	63%	0.33
Analgesics	18%	23%	
Combination	19%	13%	

Elder patients were more likely to receive further evaluation with the use of abdominal US (34.9% vs. 21.9%, P < 0.05) and CAT scan (5.2% vs. 0.7% P < 0.05). Furthermore, compared to non-elder patients, elder patients had higher referral rates for hospitalization (42.8% vs. 22.2%, P < 0.05), with the majority of admissions being in the internal medicine department and had higher length of stay (5.3 vs. 4.3 days, P < 0.05) (Table 3).

**Table 3:** Discharge Status Stratified by Age

	< 65	≥ 65	P-value
Discharge	79%	57%	0.00
Admission	22%	43%	
Department			0.00
Surgical	76%	38%	
Internal Medicine	24%	62%	
Length of stay	4.3 ± 3.1	5.3 ± 3.4	0.03
Surgery			0.96
No	91%	92%	
Yes	9%	8%	
Non-specific abdominal pain			0.07
No	84%	92%	
Yes	16%	8%	

From those patients who were admitted in the surgical department no statistically significant difference were detected regarding the necessity for emergency surgery. Moreover, the elder group had a trend towards lower cases of non-specific abdominal pain in comparison with the non-elders, nonetheless this difference was not statistically significant (P = 0.07). Between male and female elders no statistically significant differences were detected with respect to clinical presentation, laboratory values, use of imaging studies, treatment modalities, and discharge status.

## Discussion

Acute abdominal pain is one of the most common symptoms of ED patients. Differentiating severe abdominal pathology, which requires surgical intervention, from other benign conditions can be a time consuming and difficult endeavor. The clinical assessment of acute non-traumatic abdominal pain is challenging because its causes are broad, ranging from minor, self-limiting conditions to catastrophic, life-threatening diseases. Patients often have atypical historical and physical examination findings. The puzzling task of treating a patient with abdominal pain becomes even more complicated when an elderly is involved.

In this study, although no differences were detected regarding the clinical presentation and management, older patients with abdominal pain had a higher likelihood of being admitted and were more often submitted for further diagnostic tests. Previous researchers have also highlighted the necessity for more thorough work-up in elderly patients with abdominal pain [8, 9, 12]. This need rises from the fact that the older patients are more likely to suffer from severe and possible life threatening conditions such as mesenteric ischemia, ruptured abdominal aortic aneurysm, and incarcerated hernia. Furthermore, the functional status of these patients may decline very rapidly even over relatively minor symptoms owing to their impaired immunity. The latter is a result of advancing age and possible co-morbidities like diabetes mellitus and cancer. This observation denotes that the use of a more thorough work-up in the elderly group is of paramount importance in terms of dealing with diagnostic difficulties rising from atypical presentation and the presence of co-morbidities. In concordance with these observations, a study by Esses et al [13] concluded that the use of CAT scan had the ability to significantly alter clinically important decisions in elderly patients with abdominal pain.

The elder group included a higher percentage of native patients with health insurance. This finding is consistent with the fact that the majority of foreigners in our country are economic refugees of low socioeconomic status with no health insurance. What we found more interesting was that the socioeconomic status of the patients did not alter the decision making process or the referral rates. Public health is a public good and must be accessible to all people without discrimination.

Gender-based differences have been described in the elderly population in many acute medical conditions such as myocardial infarction and stroke [14, 15]. In agreement with previous researchers we failed to provide with such results [16]. We noted no difference in management and diagnoses between older men and women who presented with abdominal pain.

### Study limitations

There are several important limitations that must be addressed. Firstly, we may have lacked the power to see a wide variety of admission parameters and assess the existence of potential differences between elderly and non-elderly patients concerning the evaluation, management, and diagnosis of the non-traumatic abdominal pain in the emergency setting. Secondly, we do not have information on whether patients were admitted to a different hospital after being discharged. Furthermore, the study was conducted at a regional hospital, and the findings in these patients may not safely apply for other healthcare settings. Finally, we also did not control for representation of all geographic regions.

### Conclusions

Abdominal pain remains one of the most common complaints in the ED. A careful history and physical examination as well as a high index of suspicion are crucial to prevent missed diagnoses. A thorough work-up including blood tests, electrocardiogram, and x-ray is essential for all patients. The clinician should always be mindful that elderly patients may require more tests like U/S and CAT scan. We should not be to haste to discharge older patients, especially when we have failed to specify the origin of the abdominal pain, as, in the majority of cases, its etiology is non-specific and self-restrained.
